# Prediction of anastomotic leakage after anterior rectal resection

**DOI:** 10.12669/pjms.35.3.252

**Published:** 2019

**Authors:** Shubang Cheng, Bolin He, Xueyi Zeng

**Affiliations:** 1 *Dr. Shubang Cheng, MD, Department of Gastrointestinal, People’s Hospital of Longhua District, Affiliated Hospital of Guangdong Medical University, Shenzhen, Guangdong Province, China*; 2 *Dr. Bolin He, MD, Department of Gastrointestinal, People’s Hospital of Longhua District, Affiliated Hospital of Guangdong Medical University, Shenzhen, Guangdong Province, China*; 3 *Dr. Xueyi Zeng, MD, Department of Gastrointestinal, People’s Hospital of Longhua District, Affiliated Hospital of Guangdong Medical University, Shenzhen, Guangdong Province, China*

**Keywords:** Anastomotic leakage, LASSO, Prediction, Rectal cancer

## Abstract

**Objective:**

Anastomotic Leakage (AL) is one of the most common complications after resection of rectal cancer. Recognition of the incidence and risk factors related to AL is important. This study aimed develops a model that can predict anastomotic leakage after anterior rectal resection.

**Methods:**

Data from 188 patients undergoing anterior resection of rectal cancer were collected for retrospective analysis. Patients were randomly divided in the development set and validation set at a 1:1 ratio. We first included age, sex, preoperative chemoradiotherapy, tumor size, degree of tumor differentiation, stage, TNM stage, lymph vascular invasion, distance, anastomotic method, diabetes, intraoperative time, intraoperative bleeding and smoking as candidates for variable selection with a LASSO method. A ROC curve was constructed with the validation set to assess the accuracy of the prediction model.

**Results:**

AL occurred in 20 of 188 patients (10.6%). Preoperative chemoradiotherapy (p=0.04), medium degree of tumor differentiation (p=0.04), anastomotic method (p<0.01), intraoperative bleeding≥400ml (p<0.01), smoking (p<0.01), diabetes (p<0.01) were significantly related to AL. The area under the ROC curve of the prediction model is 0.952.

**Conclusions:**

This study developed a model that can predict anastomotic leakage after anterior rectal resection, which may aid the selection of preventive ileostomy and postoperative management.

## INTRODUCTION

Rectal cancer is the third most common malignant tumor in the world. The incidence in China has been increasing at a rate of 4.2% per year.^1^ Surgery has been a common treatment approach for rectal cancer. But anastomotic leakage (AL) is one of the most frequent complications after the resection of rectal tumors. The incidence of postoperative AL in rectal cancer is 3%-13%.^2^,^3^ It can cause serious consequences such as pelvic abscesses, peritonitis, sepsis, septic shock and even death. Moreover, AL is also the main course of long-term complications such as postoperative anastomotic stenosis and bowel dysfunction. Although significant progress has been made in the stapler, operation technique and perioperative management in recent ten years, anastomotic leakage remains a major issue in clinical practice.

Many factors^4^ are believed to affect the anastomosis, such as age, sex, level of anastomosis, ASA score, BMI, steroid treatment, preoperative chemoradiotherapy, tumor stage, tumor size, operative time, number of stapler firings, weight loss, malnutrition, fluid/electrolyte disorders, alcohol consumption, intra-operative transfusions/blood loss, smoking and diabetes but the pathogenesis remains unclear, which brings difficulties to accurately predict AL in rectal cancer. Thus colorectal surgeons may have to empirically use preventive colostomy for patients.

In this study, we aimed to develop a risk model to predict the occurrence of postoperative AL and aid the proper selection of preventive ileostomy.

## METHODS

This study was approved by the Institutional Review Board (IRB) of People’s Hospital of Longhua District (No. LW-20170301-003-01). The IRB waived the written informed consent from patients since this study was carried out retrospectively.

Patients with rectal cancer who underwent surgical treatment in our hospital from 2010 to 2016 were enrolled. Rectal cancer was defined as a tumor located at 15 cm or less from the anal verge, as determined by endoscopy and/or digital rectal examination. The inclusion criteria were patients undergoing DIXON surgery without preventive colostomy.

### Exclusion criteria were

1. Patients with colon cancer at the descending colon or above the descending colon
2. Patients undergoing Miles surgery
3. Patients undergoing Hartman surgery
4. Patients undergoing transanal or transsacral local excision of rectal cancer.

Finally, 188 patients were included, and amongst them 20 had postoperative anastomotic leakage. The diagnosis of AL was based on clinical manifestations and imaging results.

For middle and low rectal cancer, the operation was strictly restricted to the procedure of total mesorectal excision (TME). Tumor-specific mesorectal excision (TSME) was performed for the upper rectal cancer and when the mesorectal excision level was 5 cm from the lower edge of the tumor.^5^,^6^ The mode of anastomosis includes stapled anastomosis and manual anastomosis. After the anastomosis, the leak test was performed. The conventional indwelling duration of drainage tube was 7-15 days.

### Statistical analysis

Statistical analysis was conducted in R. Mean (standard deviation) and frequency (percentage) were summarized by anastomotic states for continuous and categorical variables, respectively. Chi-square tests or Fisher Exact tests were used for comparison of categorical variables and the Wilcoxon tests were used for comparison of real-value variables between the AL and non-AL groups. Half of the patients in each group were randomly selected as a training set to develop a prediction model with the LASSO method.^7^ The remaining cases were used as a validation set.

## RESULTS

A total of 188 patients were included and 20 patients including 15 males and four females had postoperative AL with an incidence rate of 10.6%. The patient-related variables, tumor-related variables, and surgery-related variables were selected for univariate analysis (Table-I) and the degree of tumor differentiation, the distance between the lower edge of the tumor and the anal margin, anastomotic method, diabetes, intraoperative time, intraoperative bleeding and smoking history were significantly associated with AL (p <0.05).

**Table-I:** Univariate analysis of risk factors for anastomotic leakage.

Variable	Overall	No Anastomotic Leak (n=168)	Anastomotic Leak (n=20)	P-value
Age	61.11±14.02	61.66(13.71)	56.45(16.06)	0.2152
***Sex***				
Female	72(38.29)	68(40.48)	4(20)	0.09
Male	116(61.70)	100(59.52)	16(80)	
***Preoperative chemoradiotherapy***				
N	174(92.55)	156(92.86)	18(90)	0.648
Y	14(7.45)	12(7.14)	2(10)	
***Tumor size***				
<4cm	72(38.29)	66(39.29)	6(30)	0.5726
≥4cm	116(61.70)	102(60.71)	14(70)	
***Degree of tumor differentiation***				
High differentiation	11(5.85)	7(4.17)	4(20)	8.24E-06
Medium differentiation	161(85.6)	152(90.48)	9(45)	
Low differentiation	16(8.51)	9(5.36)	7(35)	
***Stage***				
T1+T2	36(19.15)	34(20.24)	2(10)	0.375
T3+T4	152(80.85)	134(79.76)	18(90)	
***TNM stage***				
0	1(5)	1(0.6)	0	0.1422
I	30(15.96)	29(17.26)	1(5)	
II	64(34.04)	60(35.71)	4(20)	
III	81(43.09)	67(39.88)	14(70)	
IV	12(6.38)	11(6.55)	1(5)	
***Lymph vascular invasion***				
N	97(51.60)	88(52.38)	9(45)	0.6982
Y	91(48.40)	80(47.62)	11(55)	
***Distance***				
<7cm	51(27.13)	38(22.62)	13(65)	0.0001674
≥7cm	137(72.87)	130(77.38)	7(35)	
***Anastomotic method***				
Hand-sewn	3(1.60)	1(0.6)	2(10)	0.03033
Stapler	185(98.40)	167(99.40)	18(90)	
***Diabetes***				
N	156(82.98)	149(88.69)	7(35)	3.29E-07
Y	32(17.02)	19(11.31)	13(65)	
***Intraoperative time***				
<3.5h	136(72.34)	127(75.60)	9(45)	0.006974
≥3.5h	52(27.66)	41(24.40)	11(55)	
***Intraoperative bleeding***				
<400ml	170(90.43)	162(96.43)	8(40)	6.34E-10
≥400ml	18(9.57)	6(3.57)	12(60)	
***Smoking***				
N	146(77.66)	138(82.14)	8(40)	0.0001219
Y	42(22.34)	30(17.86)	12(60)	

Data are presented as mean ± SD or No. (%); Distance: distance between the lower edge of tumor and the anal margin

**Fig.1:**
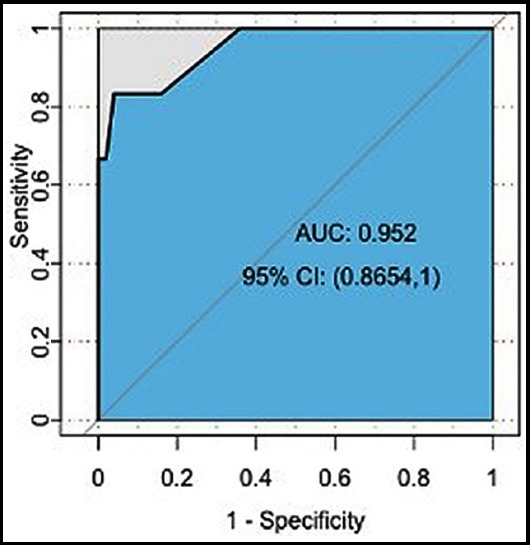
The ROC for occurrence of anastomotic leak prediction model.

Then the factors including preoperative chemoradiotherapy, degree of tumor differentiation, anastomotic method, distance, intraoperative bleeding, smoking and diabetes were selected for multivariable analysis (Table-II). The significant risk factors for AL were preoperative chemoradiotherapy (p=0.04), medium degree of tumor differentiation (p=0.04), anastomotic method (p<0.01), intraoperative bleeding more than 400ml (p<0.01), smoking (p<0.01), and diabetes (p<0.01). The validation set was used to construct the ROC curve (Fig.1) and the area under the curve (AUC) of the ROC was 0.952, indicating a good performance of the prediction model.

**Table-II:** Multivariable analysis of risk factors for anastomotic leakage

Factors	OR	Confidence interval	P-value
Preoperative chemoradiotherapy (Y)	21.01865	1.17~784.88	0.045
Degree of tumor differentiation (Medium)	0.02	1.67E-04~0.72	0.040
Degree of tumor differentiation (Low)	0.24	1.31E-03~20.63	0.542
Anastomotic method (Stapler)	5.74E-04	6.36E-07~0.08	0.007
Distance(≥7cm)	0.30	0.02~3.11	0.321
Intraoperative bleeding (≥400ml)	124.39	10.76~5403.72	0.001
Smoking (Y)	179.80	14.03~8337.25	<0.001
Diabetes (Y)	301.02	23.70~1722.13	<0.001

## DISCUSSION

AL can potentially affect short period recurrences and long-term survival.^8^,^9^ Therefore, in this study, we aimed to develop a risk model to predict the occurrence of postoperative anastomotic leakage, to guide the surgeons to select the preventive ileostomy in a more scientific and standardized way.

Despite the conspicuous progress in rectal cancer surgery, about 10.6% of the patients still have AL in our study, within the previously reported range of incidence rate (3%-13%). To select appropriate predictors, adaptive LASSO (least absolute shrinkage and selection operator) was used. LASSO method was originally developed for variable selection in building regression models and, Zou upgraded the LASSO to adaptive LASSO so that the selected model will be very close to the true models (Oracle property) as the sample size increases.^7^ The adaptive LASSO employs the appropriate penalty term to allow some of the coefficients to be estimated as zeros (variable exclusion) which is called sparsity regression. Unlike the stepwise regression using multiple steps and subjectively selected cutoff value of the p-value for exclusion, the selection of the predictors using adaptive LASSO is simultaneously achieved and the tuning parameter selection is data-driven.

We found that preoperative chemoradiotherapy, diabetes, smoking, the amount of intraoperative bleeding, anastomotic method, and degree of tumor differentiation were independent AL risk factors after resection of rectal cancer. Consistently, preoperative chemoradiotherapy for rectal cancer has been previously reported as a risk factor for AL.^10^

The histological changes of blood vessels and epithelial tissue induced by preoperative chemoradiotherapy give rise to the destruction of mucosal inflammation and mucosal barriers,^11^ which may lead to mucosal atrophy, fibrosis of the intestinal wall and hardening of the blood vessels, making the use of the stapler difficult and increasing the incidence of AL. But future studies are still needed to investigate the impact of preoperative chemoradiotherapy on AL as some reports also show that preoperative chemoradiotherapy does not affect the technical feasibility of the stapler and does not significantly increase the morbidity of postoperative complications such as AL.^12^,^13^

Smoking has been reported to affect the healing of anastomotic stoma in gastrointestinal surgery.^14^ Nicotine affects arterial smooth muscle and slows blood flow. Inhalation of carbon monoxide combined with hemoglobin, resulting in the ability of blood to carry oxygen down, affecting blood coagulation and tissue remodeling. Smoking may change the use of Nitrous Oxide in the blood, and produce blood vessels destruction. In terms of the blood itself, smoking can cause changes in the function of the blood circulation system, cause insufficient blood supplied and impede the healing of the wound. On the other hand, smoking may alter the mechanism of inflammation, reduce the influx of macrophages in the inflammatory response, and reduce the formation of proinflammatory factors that control adhesion and migration. Vignali A et al.^2^ reported a correlation between diabetes mellitus and AL. Diabetic patients with systemic metabolic disorder and hyperglycemia lead to large amounts of reactive oxygen species (ROS) and advanced glycation end products (AGEs),^15^ which damage microvascular endothelial cells, resulting in abnormal flow of microvascular endothelial cells. Lipid metabolism and glucose metabolism disorder coexist in diabetic patients. The damage mechanism of lipotoxicity on microvascular endothelial cells is the increasing production of reactive oxygen species (ROS) caused by the up regulation of the expression level of the NADPH oxidase complex active functional subunit.^16^ The damage of the anastomotic microvessels hinders the healing of the anastomotic stoma.

Hand-sewn was also found to be a risk factor of anastomotic leakage. There was significant difference (p < 0.05) in AL between stapled and hand-sewn colorectal anastomosis. A Cochrane review of colorectal anastomosis after right hemicolectomy showed that the total anastomotic leakage rate in the stapler group was significantly reduced.^17^ An interesting subgroup analysis conducted by Friend PJ et al.^18^ found that there was more anastomotic leakage in hand-sewn colorectal anastomosis when analysing the anastomosis alone. Their conclusion is that stapler anastomosis seems to be more suitable for surgeons without numerous surgical experience. The double stapling technique reduces the difficulty of the operation and reduces the difficulty of anastomosis due to the inconsistent caliber of the bowel lumen at both ends. Moreover, the design of the nail spacing, the row spacing and the varus degree of the intestinal wall by the stapler was more accurate and reasonable than manual suture. It can effectively avoid overflow of intestinal contents and reduce the chance of infection around the anastomotic stoma as well as the pelvic cavity. So the incidence of anastomotic leakage can be reduced.

Wang L and Gu J^19^ reported the risk factor for symptomatic AL after anterior resection of low rectal cancer. Their results showed that blood loss more than 200 ml was identified as independent risk factors for AL. A systematic review by McDermott FD et al.^20^ showed that blood loss more than 100 ml was an independent risk factor for AL. In contract, Crombe T et al.^21^ reported that blood loss more than 500 ml did not significantly increase the risk of AL. In our study, intraoperative bleeding more than 400 ml was significantly associated with the increased risk of AL. This result suggests that prophylactic stoma should be considered when the amount of bleeding is large during the operation.

Although there have been several studies^22^,^23^ reported that T staging is associated with the occurrence of AL, no studies have addressed the relationship between AL and the degree of tumor differentiation. Our study shows that the degree of tumor differentiation is also a risk factor of anastomotic leakage after anterior resection of rectal cancer.

Low anastomosis is the most important risk factor for AL.^24^,^25^ In a previous study^24^, anal verge distance <7 cm was a risk factor associated with AL occurrence. A study conducted by Vignali A et al.^2^ showed that the incidence of anastomotic leakage was 8% when the lower edge of the tumor was within 7 cm from the anal margin, and when the tumor’s lower margin was more than 7 cm from the anal margin, it was 1%. Pakkastie TE et al.^25^ described similar results. They also determined the difference between high and low anastomosis at a distance of 7 cm from the edge of the anus. The high leakage rate associated with low anastomosis is probably due to the location of the tumor was lower, and that the free range of rectal surgery is greater, which increase the difficulty of the anastomosis with increased anastomotic tension and decrease the rectal blood supply. So it affects local healing and anti-infection ability, and increases the chance of bacterial infection, resulting in an increased chance of AL. However, our study found that Distance (≥7cm) was not protective factor for AL (p=0.32). Future studies are required to investigate feasibility of the selection of 7 cm as a cutoff point.

### Limitations of study

This was a single center study, which result in sample bias. This prediction model could not define the actual situation of AL according to current risk factors. We expect that a prospective large-sample-size and multicenter study could be conducted in future to improve the reliability and practicability of the prediction model.

## CONCLUSION

We have developed a model to predict AL after anterior rectal resection by using the LASSO method with an AUC 0.952. Preoperative chemoradiotherapy, diabetes, smoking, intraoperative bleeding, degree of tumor differentiation and method of anastomosis are independent risk factors for AL.

### Author`s Contribution

**SC** conceived and designed the study.

**SC, BH and XZ** did data collection and manuscript writing.

**SC and XZ** did statistical analysis.

**SC, BH and XZ** did review and final approval of manuscript.
